# Outcomes of non-high grade serous carcinoma after neoadjuvant chemotherapy for advanced-stage ovarian cancer: a Korean gynecologic oncology group study (OV 1708)

**DOI:** 10.1186/s12885-019-5514-7

**Published:** 2019-04-11

**Authors:** Young Shin Chung, Sang-Yoon Park, Jung-Yun Lee, Jeong-Yeol Park, Jeong-Won Lee, Hee Seung Kim, Dong Soo Suh, Yun Hwan Kim, Jong-Min Lee, Miseon Kim, Min Chul Choi, Seung-Hyuk Shim, Keun Ho Lee, Taejong Song, Jin Hwa Hong, Won Moo Lee, Banghyun Lee, In Ho Lee

**Affiliations:** 1 Department of Obstetrics and Gynecology, Institute of Women’s Life Medical Science, Yonsei University College of Medicine, 50-1 Yonsei-ro, Seodaemun-gu, Seoul, 03722 South Korea; 2 Center for Uterine Cancer, Hospital, National Cancer Center, 323 Ilsan-ro, Ilnsandong-gu, Goyang-si, Gyeonggi-do 410-769 South Korea; 3 Department of Obstetrics and Gynecology, University of Ulsan College of Medicine, Asan Medical Center, Seoul, South Korea; 4 Department of Obstetrics and Gynecology, Samsung Medical Center, Sungkyunkwan University School of Medicine, Seoul, South Korea; 5 Department of Obstetrics and Gynecology, Seoul National University College of Medicine, Seoul, South Korea; 6 Department of Obstetrics and Gynecology, Pusan National University School of Medicine, and Biomedical Research Institute, Pusan National University Hospital, Busan, South Korea; 7 Division of Gynecologic Oncology, Department of Obstetrics and Gynecology, Ewha Womans University College of Medicine, Seoul, South Korea; 8 Department of Obstetrics and Gynecology, Kyung Hee University Hospital at Gangdong, Kyung Hee University College of Medicine, Seoul, South Korea; 9 Department of Obstetrics and Gynecology, CHA Gangnam Medical Center, CHA University College of Medicine, Seoul, South Korea; 10 Comprehensive Gynecologic Cancer Center, CHA Bundang Medical Center, CHA University, Seongnam, South Korea; 11 Department of Obstetrics and Gynecology, Konkuk University School of Medicine, Seoul, South Korea; 12 Department of Obstetrics and Gynecology, Seoul St. Mary’s Hospital, College of Medicine, The Catholic University of Korea, Seoul, South Korea; 13 Department of Obstetrics and Gynecology, Kangbuk Samsung Hospital, Sungkyunkwan University School of Medicine, Seoul, South Korea; 14 Department of Obstetrics and Gynecology, Guro Hospital, College of Medicine, Korea University, Seoul, South Korea; 15 Department of Obstetrics and Gynecology, Hanyang University College of Medicine, Seoul, South Korea; 16 Department of Obstetrics and Gynecology, Hallym University Kangdong Sacred Heart Hospital, Seoul, South Korea; 17 Department of Obstetrics and Gynecology, Cheil General Hospital and Women’s Healthcare Center, Dankook University College of Medicine, Seoul, South Korea

**Keywords:** Ovarian neoplasms, Histologic subtype, Neoadjuvant therapy, Non-high grade serous carcinoma, Survival

## Abstract

**Background:**

Outcomes of patients with ovarian high-grade serous carcinoma (HGSC) treated with neoadjuvant chemotherapy (NAC) have been widely studied, but there is limited information on the outcomes of patients with non-HGSC. This study aimed to evaluate the outcomes of NAC in non-HGSC patients with advanced-stage ovarian cancer.

**Methods:**

We conducted a retrospective cohort study of patients who underwent NAC for advanced stage non-HGSC between 2002 and 2017 in 17 institutions. Demographics, surgical outcomes, and survival rates were evaluated according to histological subtypes.

**Results:**

A total of 154 patients were included in this study, comprising 20 cases (13.0%) of mucinous adenocarcinoma, 31 cases (20.1%) of endometrioid adenocarcinoma, 28 (18.2%) cases of clear cell carcinoma, 29 (18.8%) cases of low-grade serous carcinoma and 12 cases (7.8%) of carcinosarcoma. Complete remission/partial remission after the third cycle of NAC was achieved in 100 (64.9%) patients and optimal debulking surgery (residual disease ≤1 cm) at interval debulking surgery was achieved in 103 (66.9%) patients. The most common reason for performing NAC was high tumor burden (*n* = 106, 68.8%). The median progression-free survival (PFS) was 14.3 months and median overall survival (OS) was 52.9 months. In multivariate analyses, mucinous and clear cell carcinoma were negative prognostic factors for both PFS (*p* = 0.007 and *p* = 0.017, respectively) and OS (*p* = 0.002 and *p* = 0.013, respectively).

**Conclusions:**

In this study, poor survival outcomes were observed in patients with mucinous and clear cell carcinoma undergoing NAC. Different treatment strategies are urgently required to improve survival outcomes for this disease subset.

**Electronic supplementary material:**

The online version of this article (10.1186/s12885-019-5514-7) contains supplementary material, which is available to authorized users.

## Background

Ovarian cancer is the leading cause of death from gynecologic malignancy worldwide [1]. In Korea, the incidence and mortality rates of ovarian cancer are steadily increasing [2–4]. The standard treatment for advanced-stage ovarian cancer is cytoreductive surgery followed by platinum-based combination chemotherapy [5, 6]. Despite an initial response to standard treatment, the overall 5-year survival rate of advanced-stage ovarian cancer is approximately 30% [7]. However, there has been no significant change in the accepted approach to treatment during the last two decades, which has hampered improvements in long-term survival [8].

In part, this is due to epithelial ovarian cancer being considered as a heterogeneous disease across different histologic subtypes, including high-grade serous carcinoma (HGSC), clear cell carcinoma, endometrioid, mucinous carcinoma, and low-grade serous carcinoma (LGSC) [9]. Since 1999, the incidence of clear cell carcinoma has increased markedly across all age groups in Korea [10]. Furthermore, histologic subtypes other than HGSC (non-HGSC) are known to have poorer clinical outcomes in response to conventional chemotherapy due to resistance and reduced sensitivity of these subtypes to chemotherapy [11–15]. Previous clinical data have indicated that survival outcome correlates with the histological subtype [11]. However, all ovarian cancers continue to be treated with the same therapeutic strategy regardless of subtype.

Some previous studies showed that patients with non-HGSC had poorer survival than those with HGSC in primary debulking surgery (PDS) [14–18]. There are several randomized studies showing that neoadjuvant chemotherapy (NAC) is non-inferior to PDS for patients with advanced epithelial ovarian cancer [19–21]. However, NAC has primarily been evaluated by studies focusing on HGSC [22] and currently there are no published studies on NAC outcomes in patients with non-HGSC. Furthermore, although non-HGSC is considered a different disease entity than HGSC, it is not yet known whether the selection criteria for NAC and the treatment regimen of NAC should be the same for patients with non-HGSC as those for patients with HGSC.

Therefore, we conducted a multicenter retrospective cohort study to evaluate the clinical, surgical and survival outcomes of non-HGSC patients with advanced-stage ovarian cancer after NAC.

## Methods

From 2002 to 2017, 154 patients who had undergone NAC for advanced stage non-HGSC were enrolled from 17 institutions affiliated with the Korean Gynecologic Oncology Group (KGOG). We showed the number of enrolled patients from each institution in Additional file 1: Table S1. We performed a retrospective cohort study and data were collected from medical records. This study was approved by the institutional review boards of the participating centers in accordance with the Declaration of Helsinki and the International Conference of Harmonization Good Clinical Practice Guidelines.

Inclusion criteria for patients were as follows: (1) diagnosed and histologically confirmed with non-HGSC subtype of epithelial ovarian cancer; (2) diagnosed with International Federation of Gynecology and Obstetrics (FIGO) stage III or IV; (3) received at least 1 cycle of NAC with or without interval debulking surgery (IDS); and (4) had available medical records and follow-up data. All cases diagnosed as non-HGSC subtype by cytologic evaluation of ascites/pleural effusion, image-guided aspiration biopsy, diagnostic laparoscopic/laparotomy biopsy, or after IDS were included. The exclusion criteria were as follows: (1) non-epithelial ovarian cancer; (2) HGSC; (3) FIGO stage I or II; (4) borderline epithelial ovarian cancer; and (5) PDS as primary therapy.

Reasons for the use of NAC varied due to the long-term, multicenter, retrospective nature of this analysis. Therefore, reasons for the use of NAC were collected from each patient.

Clinical staging of epithelial ovarian cancer was performed according to the FIGO system (2014) following the use of computed tomography (CT) and positron emission tomography (PET)/CT for preoperative imaging workup. Response to chemotherapy was evaluated after the third cycle of NAC using the Response Evaluation Criteria in Solid Tumors (RECIST) criteria [23].

All patients received NAC as primary therapy with various regimens in accordance with institutional policy. Standard surgical procedure consisted of total hysterectomy, bilateral salpingo-oophorectomy, omentectomy, or retroperitoneal lymphadenectomy. Radical surgeries were defined as bowel resection, diaphragm/peritoneal surface stripping, splenectomy, liver resection, partial gastrectomy, or partial cystectomy/ureteroneocystostomy. Postoperative complications were graded according to the Memorial Sloan-Kettering Cancer Center’s (MSKCC) surgical secondary events grading system [24]. We defined the major surgical complications as MSKCC grade ≥ 3. After surgery, patients underwent various cycles and regimens of postoperative adjuvant chemotherapy (POAC). Histologic subtypes were confirmed according to the criteria of the World Health Organization classification; however, there was no central review of all microscopic slides. After the completion of primary treatment, all patients underwent institutional routine clinical follow-up, consisting of imaging studies and blood tests. During follow-up, clinical assessment including pelvic examination, serum CA-125 level were every 2–3 months, and imaging studies such as contrast-enhanced CT scan was performed every 6 months.

### Statistical analysis

Continuous variables were described as median and range (minimum and maximum value), while categorical data were described by frequencies and percentages (%). Progression-free survival (PFS) was defined as the time from the date of first NAC to the date of first recurrence or the date of last follow-up. Recurrence or progression was diagnosed by radiologic finding, but not by elevated CA-125 level alone. Overall survival (OS) was defined as the time from the date of first NAC to the date of death or the date of last follow-up. At each institution, survival data were extracted from their own cancer registry linked with death certificate. Kaplan-Meier survival curves were calculated using the log-rank test. Univariate and multivariate analyses were evaluated using a Cox proportional hazard model, and prognostic factors affecting PFS and OS were evaluated. A *p*-value < 0.05 was considered to indicate statistical significance. Data were analyzed with IBM SPSS, version 23 for Windows (IBM Corp, Armonk, NY, USA).

## Results

Baseline characteristics are shown in Table 1. Of the enrolled 154 patients, 20 patients (13.0%) were diagnosed with mucinous adenocarcinoma; 31 (20.1%) with endometrioid adenocarcinoma; 28 (18.2%) with clear cell carcinoma; 29 (18.8%) with LGSC; and 12 (7.8%) with carcinosarcoma. Three patients did not undergo IDS and 8 patients did not receive POAC. The median number of NAC cycles, POAC cycles, and total chemotherapy cycles were 3 (range: 1–10), 6 (range: 0–22), and 9 (range: 2–28), respectively. Most of patients treated with a platinum-taxane combination of NAC (*n* = 152, 98.7%) or POAC (*n* = 129, 83.6%). Various methods of diagnosis were used before NAC: cytology of ascites in 59 cases (38.3%), diagnostic laparoscopy/laparotomy in 41 cases (26.6%), aspiration biopsy in 31 cases (20.1%), and cytology of pleural effusion in 14 cases (9.1%). However, histologic subtypes were confirmed before NAC in only 46 cases (*n* = 29.9%). Therefore, in some cases NAC was administered regardless of histologic subtype and according to criteria such as high tumor burden, older age, or poor Eastern Cooperative Oncology Group (ECOG). The reasons (multi-selectable) for NAC in patients with non-HGSC were high tumor burden (*n* = 106, 68.8%), old age/poor ECOG (*n* = 24, 15.6%), considered as HGSC before NAC (*n* = 8, 5.2%), being referred from another hospital after NAC (*n* = 5, 3.3%), and others such as policy of each institution (*n* = 30, 19.5%). NAC was obtained in 13 patients (8.4%) with both high tumor burden and old age/poor ECOG.

**Table 1:** Baseline characteristics of patients (*n* = 154)

Characteristics	N (%)
Median age, years (range)	54.0 (27.0–79.0)
Median BMI at diagnosis, kg/m^2^ (range)	22.5 (12.9–31.9)
Median baseline CA-125 level, U/mL (range)	808.0 (9.0–19,800.0)
FIGO stage, n (%)	
III	68 (44.2)
IV	86 (55.8)
ASA score, n (%)	
1–2	122 (79.2)
3–4	21 (13.6)
Unknown	11 (7.2)
Histologic subtype, n (%)	
Mucinous	20 (13.0)
Endometrioid	31 (20.1)
Clear cell	28 (18.2)
LGSC	30 (19.5)
Carcinosarcoma	12 (7.8)
Undifferentiated	20 (13.0)
Mixed	5 (3.3)
Anaplastic	2 (1.3)
Transitional	3 (2.0)
Squamous cell carcinoma	2 (1.3)
Ewing’s sarcoma	1 (0.6)
Method of diagnosis, n (%)	
Ascites cytology	59 (38.3)
Pleural effusion cytology	14 (9.1)
Aspiration biopsy	31 (20.1)
Diagnostic laparoscopy	24 (15.6)
Diagnostic laparotomy	4 (2.6)
Laparoscopy in other hospital	12 (7.8)
Laparotomy in other hospital	1 (0.6)
Others	5 (3.3)
Not diagnosed before NAC	4 (2.6)
Confirm the histologic subtype before NAC, n (%)	
No	108 (70.1)
Yes	46 (29.9)
HGSC	5 (3.3)
Mucinous	7 (4.6)
Endometrioid	4 (2.6)
Clear cell	8 (5.2)
LGSC	9 (5.9)
Carcinosarcoma	2 (1.3)
Others	11 (7.1)
Reasons for performing NAC^*^, n (%)	
Old age / poor ECOG	24 (15.6)
High tumor burden	106 (68.8)
Considered as HGSC before NAC	8 (5.2)
Refer from other hospital after NAC	5 (3.3)
Others	30 (19.5)
NAC regimens, n (%)	
Paclitaxel + carboplatin	137 (89.0)
Paclitaxel + cisplatin	6 (3.9)
Docetaxel + carboplatin	8 (5.2)
Paclitaxel + carboplatin + bevacizumab	1 (0.6)
Others	2 (1.3)
POAC regimens, n (%)	
Paclitaxel + carboplatin	114 (74.0)
Paclitaxel + cisplatin	5 (3.2)
Docetaxel + carboplatin	9 (5.8)
Paclitaxel + carboplatin + bevacizumab	1 (0.6)
Others	17 (11.0)
Not done	8 (5.2)

*BMI* body mass index, *CA* 125 cancer antigen 125, *FIGO* Federation of Gynecology and Obstetrics, *ASA* American Society of Anesthesiologists, *NAC* neoadjuvant chemotherapy, *POAC* postoperative adjuvant chemotherapy, *HGSC* high-grade serous carcinoma, *LGSC* low-grade serous carcinoma, *ECOG* Eastern Cooperative Oncology Group

^*^Multi-selectable

The median follow-up duration was 20.3 months (range: 0.7–98.0 months), during which there were 107 recurrences and 56 deaths. Table 2 shows treatment outcomes after NAC and surgical outcomes. In response to NAC, decrease of CA-125 levels by more than 90% and normalization of CA-125 levels (< 35 U/mL) from baseline to after the third cycle of NAC were observed in 61 (39.6%) and 49 (31.8) patients, respectively. After the third cycle of NAC, 100 (64.9%) patients showed complete remission or partial remission based on RECIST criteria. However, we could not know the response rate after the third cycle of NAC of 26 patients who received less than 3 cycles of NAC (*n* = 23) or did not perform any imaging workup after the third cycle of NAC (n = 3).

**Table 2:** Treatment outcomes after NAC/IDS

Characteristics	N (%)
NAC response	
CA-125, n (%)	
CA-125 after 3rd NAC < 35	49 (31.8)
CA-125 reduction rate ≥ 90%	61 (39.6)
Response rate after 3rd NAC, n (%)	
CR	2 (1.3)
PR	98 (63.6)
SD	21 (13.6)
PD	7 (4.5)
Unknown	26 (16.9)
Surgical outcome	
Surgery extent, n (%)	
Standard	82 (53.3)
Radical	69 (44.8)
Not surgery	3 (1.9)
Residual disease after IDS, n (%)	
≤1 cm	103 (66.9)
>1 cm	25 (16.2)
Unknown	26 (16.9)
Lymphadenectomy, n (%)	
(−)	36 (23.4)
(+)	115 (74.7)
Unknown	3 (1.9)
Lymph node metastasis, n (%)	
No	88 (57.2)
Yes	63 (40.9)
Unknown	3 (1.9)
Peritoneal cytology, n (%)	
Negative	57 (37.0)
Positive	63 (40.9)
Not tested	27 (17.5)
Unknown	7 (4.6)
Postoperative complications^*^	
0–2	143 (92.9)
3–5	8 (5.2)
Unknown	3 (1.9)

*NAC* neoadjuvant chemotherapy, *IDS* interval debulking surgery, *HGSC* high-grade serous carcinoma, *CA* 125 cancer antigen 125, *CR* complete response, *PR* partial response, *SD* stable disease, *PD* progressive disease

^*^According to the Memorial Sloan-Kettering Cancer Center’s surgical secondary events grading system

Regarding surgical outcomes, 103 (66.9%) patients underwent optimal debulking surgery (residual disease ≤1 cm). One hundred fifteen (74.7%) patients underwent lymphadenectomy and of these, 63 patients (40.9%) had lymph node metastasis. Sixty-three (40.9%) women had positive peritoneal cytology during IDS. Eight patients (5.2%) experienced major complications (MSKCC grade ≥ 3) related to surgery.

**Fig. 1:**
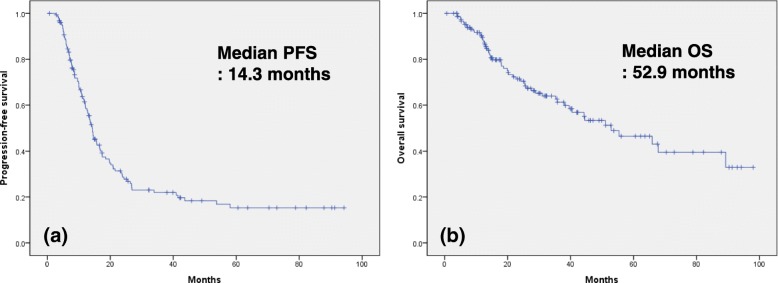
Kaplan-Meier survival curves for patients with non-HGSC. Progression-free survival (**a**). Overall survival (**b**). HGSC, high-grade serous carcinoma

**Fig. 2:**
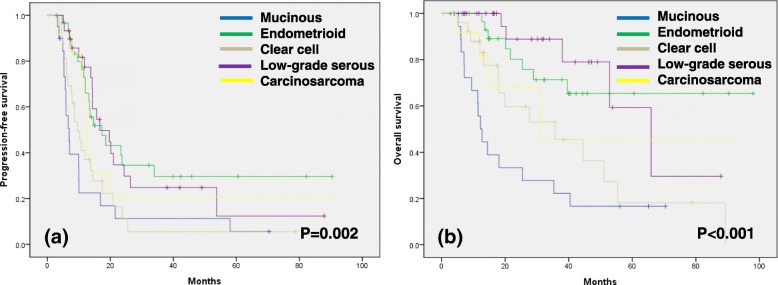
Kaplan-Meier survival curves stratified by histologic subtype. Progression-free survival (**a**). Overall survival (**b**)

**Fig. 3:**
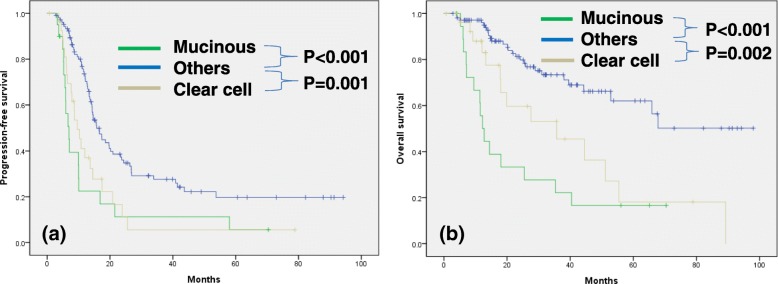
Kaplan-Meier survival curves for patients with mucinous or clear cell carcinoma compared to other subtypes. Progression-free survival (**a**). Overall survival (**b**)

Survival analysis showed that the median PFS was 14.3 months and the median OS was 52.9 months (Fig. 1). The Kaplan-Meier curves stratified by histologic subtypes are shown in Fig. 2. PFS and OS differed significantly according to histologic subtypes (PFS, *p* = 0.002; OS, *p* < 0.001). The median PFS by subtype was as follows: 6.9 months for mucinous adenocarcinoma, 17.4 months for endometrioid adenocarcinoma, 9.6 months for clear cell carcinoma, 16.6 months for LGSC, and 12.6 months for carcinosarcoma. The median OS by subtype was as follows: 12.2 months for mucinous adenocarcinoma, 35.7 months for clear cell carcinoma, 65.9 months for LGSC, and 31.0 months for carcinosarcoma, while the median OS for endometrioid adenocarcinoma was not reached. Overall, mucinous adenocarcinoma and clear cell carcinoma appeared to have the poorest survival prognosis. Multivariate Cox proportional hazards regression showed that American Society of Anesthesiologists score, residual tumor, and lymph node dissection all demonstrated an independent significant impact on PFS and OS. Furthermore, mucinous carcinoma was found to be a significantly negative predictor for both PFS (hazard ratio [HR]: 2.31, 95% confidence interval [CI]: 1.26–4.24, *p* = 0.007) and OS (HR: 3.24, 95% CI: 1.54–6.82, p = 0.002). Similarly, clear cell carcinoma also appeared to be a poor prognostic factor for both PFS (HR: 1.92, 95% CI: 1.12–3.29, *p* = 0.017) and OS (HR: 2.56, 95% CI: 1.21–5.30, *p* = 0.013) in patients who received NAC (Table 3). Survival outcomes were significantly poorer in patients with mucinous (PFS, p < 0.001; OS, p < 0.001) or clear cell subtype (PFS, *p* = 0.001; OS, p = 0.002) compared to other subtypes (Fig. 3).

**Table 3:** Univariate and multivariate analyses for progression-free and overall survival using the Cox proportional hazard model

Variables	PFS		OS	
	Univariate analysis	Multivariate analysis	Univariate analysis	Multivariate analysis
	HR (95% CI) P	HR (95% CI) P	HR (95% CI) P	HR (95% CI) P
Age (years)				
<55	1.00 (Reference)	1.00 (Reference)	1.00 (Reference)	1.00 (Reference)
≥55	0.99 (0.67–1.44) 0.939	1.33 (0.80–2.20) 0.268	1.14 (0.67–1.92) 0.634	1.51 (0.75–3.04) 0.245
ASA				
1–2	1.00 (Reference)	1.00 (Reference)	1.00 (Reference)	1.00 (Reference)
3–4	2.29 (1.37–3.84) 0.002	2.38 (1.26–4.51) 0.008	3.12 (1.64–5.90) < 0.001	3.24 (1.54–6.82) 0.009
Baseline CA-125 level (U/ml)				
≤800	1.00 (Reference)	1.00 (Reference)	1.00 (Reference)	1.00 (Reference)
>800	1.04 (0.71–1.53) 0.826	1.14 (0.69–1.90) 0.605	0.79 (0.47–1.34) 0.382	0.93 (0.45–1.91) 0.838
FIGO stage				
III	1.00 (Reference)	1.00 (Reference)	1.00 (Reference)	1.00 (Reference)
IV	0.91 (0.62–1.33) 0.629	0.74 (0.47–1.18) 0.210	0.75 (0.44–1.26) 0.277	0.62 (0.32–1.24) 0.177
Histology				
Others	1.00 (Reference)	1.00 (Reference)	1.00 (Reference)	1.00 (Reference)
Mucinous	2.64 (1.54–4.50) < 0.001	2.31 (1.26–4.24) 0.007	4.69 (2.48–8.85) < 0.001	3.24 (1.54–6.82) 0.002
Clear cell	2.10 (1.30–3.42) 0.003	1.92 (1.12–3.29) 0.017	2.62 (1.37–4.99) 0.004	2.56 (1.21–5.30) 0.013
Residual disease				
≤1 cm	1.00 (Reference)	1.00 (Reference)	1.00 (Reference)	1.00 (Reference)
>1 cm	2.86 (1.75–4.66) < 0.001	2.40 (1.43–4.04) 0.001	3.46 (1.89–6.33) < 0.001	2.43 (1.28–4.63) 0.007
Surgery extent				
Standard	1.00 (Reference)	1.00 (Reference)	1.00 (Reference)	1.00 (Reference)
Radical	0.94 (0.64–1.39) 0.771	1.75 (1.02–2.99) 0.042	0.98 (0.57–1.67) 0.933	1.86 (0.97–3.56) 0.062
LND				
No	1.00 (Reference)	1.00 (Reference)	1.00 (Reference)	1.00 (Reference)
Yes	0.67 (0.17–2.76) 0.004	0.33 (0.18–0.61) < 0.001	0.36 (0.09–1.49) 0.002	0.32 (0.16–0.62) 0.001
LN metastasis				
No	1.00 (Reference)	1.00 (Reference)	1.00 (Reference)	1.00 (Reference)
Yes	1.03 (0.25–4.26) 0.074	2.89 (1.65–5.07) < 0.001	0.46 (0.11–1.97) 0.553	1.78 (0.74–4.32) 0.201

*PFS* progression-free survival, *OS* overall survival, *HR* hazard ratio, *CI* confidence interval, *ASA* American Society of Anesthesiologists, *CA*-125 cancer antigen 125, *FIGO* Federation of Gynecology and Obstetrics, *M/C* Mucinous/Clear, *LND* lymph node dissection, *LN* lymph node

## Discussion

In this multicenter retrospective cohort study, we found that non-HGSC had a poor response rate after the third cycle of NAC, low rate of optimal debulking surgery compared to other studies mainly focused on HGSC. In the CHORUS trial [19], HGSC subtype rate was 71% and optimal debulking surgery rate was 73% and in Surgical Complications Related to Primary or Interval Debulking in Ovarian Neoplasms [21], HGSC subtype rate was 98.1%, optimal debulking surgery rate was 90.4%, and complete response/partial response rate according to RECIST was 90.9% in the NAC group.

The survival outcome in our study was not poor compared to the European Organization for Research and Treatment of Cancer (EORTC) 55,971 [20] and in the CHORUS trial. The median PFS and OS for patients were 12 and 30 months, respectively, in the EORTC study and 12 and 24.1 months, respectively, in the CHORUS trial in the NAC group. However, we found that mucinous and clear cell subtypes in our study have poor survival from survival curves stratified by histologic subtypes and they could be independent prognostic factors for survival.

Ovarian cancer is typically considered as a single disease with a notably heterogeneous group of neoplasms. In particular, the pathogenesis of non-HGSCs has been shown to be unique for each histologic subtype, and the clinical understanding of the pathology and molecular biology of each subtype continues to improve. However, the current guidelines suggest the same treatment strategies for patients with ovarian cancer regardless of subtype. The findings of the present study show that different histologic subtypes have different prognoses, and the response to standard chemotherapy is generally poor in non-HGSC. Therefore, further studies and approaches that explore individualized treatment strategies for histologic subtypes are necessary.

In the NAC setting, maximal surgical effect is excluded and sensitivity and response to NAC has been a well-known prognostic factor for survival in advanced-stage ovarian cancer. While patients with HGSC tend to have good responses to taxane-platinum combination regimens, there are limited comparable data for patients with non-HGSC. Non-HGSC is not as frequent as HGSC, and thus it can be difficult to acquire prospective data. With data of the Korean Central Cancer registry, we previously showed that no improvement of survival outcomes was observed for patients with clear cell carcinoma and mucinous carcinoma during the past 20 years [8]; additionally, it was not clear whether the selection criteria and regimen of NAC for patients with non-HGSC should be the same as those of patients with HGSC.

In the current study, confirmation of the histologic subtype before NAC was not common; indeed, the histologic subtypes of only 46 patients (29.9%) were identified before NAC. Thus, approximately 70% of the patients in our cohort received NAC without a histologic subtype evaluation. Histologic subtypes can be determined by diagnostic laparoscopy/laparotomy relatively easily, but cytologic evaluation of ascites and pleural effusions is more difficult. In our cohort, diagnostic laparoscopy/laparotomy was performed for just 41 patients (26.6%). An increased use of diagnostic laparoscopy could improve the rate of patients for whom histologic subtypes are identified prior to the decision to use treatment options such as PDS or NAC. Although we could not be sure for improved survival if patients without determining histologic subtypes underwent PDS instead of NAC, we could at least give a chance for clinical trials targeting specific molecular alterations associated with histologic subtypes. There is an unmet clinical need for novel therapeutic approaches tailored to patients with non-HGSC after NAC.

Recently, studies of specific histologic subtypes have been completed or are ongoing. For example, significantly higher levels of estrogen receptor (ER), progesterone receptor (PR), and E-cadherin have been shown to be expressed in LGSC compared to HGSC, [25] and a consideration of these differences may lead to the development of different therapeutic strategies [26–29]. Based on retrospective studies [29] showing the benefits of letrozole maintenance, MD Anderson group suggested a randomized phase III trial comparing letrozole and chemotherapy in LGSC. The MEK inhibitor (MILO) trial is a phase III study investigating the efficacy of MEK 162, a MEK inhibitor, compared to physician choice chemotherapy for recurrent or persistent LGSC. A phase III trial (Gynecologic Oncology Group (GOG)-241) compared the efficacy of chemotherapy regimens for mucinous carcinoma: capecitabine-oxaliplatin versus carboplatin-paclitaxel and with or without bevacizumab in mucinous subtype tumors was closed early for slow accrual, and neither of regimen clearly improved PFS or OS [30, 31]. Furthermore, in mucinous carcinoma, trastuzumab (Herceptin) may be effective for patients with HER2 overexpression [32].

Efforts are ongoing worldwide to identify better strategies for treating clear cell carcinoma, as conventional chemotherapy is considered to be less effective for this particular subtype. One randomized phase III trial (Japanese Gynecologic Oncology Group (JGOG) 3017) compared an irinotecan-cisplatin regimen with a conventional paclitaxel-carboplatin regimen for clear cell carcinoma, but the irinotecan-cisplatin regimen did not show a significant survival benefit [33]. Clear cell carcinoma frequently has both PIK3CA mutation (oncogene) and ARID1A mutation (tumor suppressor gene) [34]. The GOG recently completed accrual of patients for a front-line, phase II study of temsirolimus, an mTOR inhibitor, used following paclitaxel/carboplatin as a first-line therapy in treating patients with newly diagnosed stage III/IV clear cell carcinoma (GOG-268). However, studies of each non-HGSC subtypes have been evaluated only in patients who underwent PDS. There are no studies to identify better strategies or develop of novel molecular target therapy for non-HGSC subtypes in patients who underwent NAC/IDS. Therefore, further studies are needed to suggested optimal therapies for non-HGSC subtypes.

This study has several limitations. First, a central pathology review was not performed. Central pathology review could establish uniform pathologic criteria for specimens from patients used in studies by reducing interobserver variability. Furthermore, this study includes quite a number of undifferentiated subtypes with unclear specific subtype. These data could be more accurately represented by a central pathology review. Second, because this study was performed as a multicenter retrospective analysis, no uniform selection criteria for NAC were established. Selection for NAC was determined by the patient’s physical status, age, extent of the tumor burden, and policy of each institution, among other factors.

Despite these limitations, however, the current study is an important contribution to existing literature, as it is the first large retrospective analysis to evaluate the outcomes of non-HGSC patients after NAC in advanced-stage ovarian cancer.

## Conclusions

In this study, significantly poor response rates and low rate of optimal debulking surgery were observed among patients with non-HGSC subtypes. Mucinous and clear cell carcinomas in particular could be negative prognostic factors for survival. Therefore, our findings suggest that different treatment strategies are essential and further studies with addition of targeted agents based on biomarker should be incorporated with priority to improve survival outcomes of patients with this disease subset.

## Additional file

Additional file 1: **Table S1.** The list of enrolled patients from each institution (DOCX 24 kb)

## Abbreviations

CI: Confidence interval
CT: Computed tomography
ECOG: Eastern Cooperative Oncology Group
FIGO: Federation of Gynecology and Obstetrics
HGSC: High-grade serous carcinoma
HR: Hazard ratio
IDS: Interval debulking surgery
KGOG: Korean Gynecologic Oncology Group
LGSC: Low-grade serous carcinoma
MSKCC: Memorial Sloan-Kettering Cancer Center
NAC: Neoadjuvant chemotherapy
OS: Overall survival
PDS: Primary debulking surgery
PET: Positron emission tomography
PFS: Progression-free survival
POAC: Postoperative adjuvant chemotherapy
RECIST: Response Evaluation Criteria in Solid Tumors
