# Supplementary material for: Outcomes of non-high grade serous carcinoma after neoadjuvant chemotherapy for advanced-stage ovarian cancer: a Korean gynecologic oncology group study (OV 1708)
Source: BMC Cancer. 2019 Apr 11;19:341. doi: 10.1186/s12885-019-5514-7 (PMC6458623; doi:10.1186/s12885-019-5514-7)
Supplement: Supplementary file 1 — Table S1. The list of enrolled patients from each institution (DOCX 24 kb) [file 12885_2019_5514_MOESM1_ESM.docx]

Additional file 1: Table S1. The list of enrolled patients from each institution

| Institutions | N (%) | |
| --- | --- | --- |
| Kyung Hee University Gangdong Hospital | | 4 (2.6%) |
|  | |  |
| Hallym University Kangdong Sacred Heart Hospital | | 1 (0.6%) |
|  | |  |
| Sungkyunkwan University Kangbuk Samsung Hospital | | 2 (1.3%) |
|  | |  |
| Konkuk University Hospital | | 3 (1.9%) |
|  | |  |
| Korea University Guro Hospital | | 2 (1.3%) |
|  | |  |
| National Cancer Center | | 48 (31.2%) |
|  | |  |
| Pusan National University Hospital | | 6 (3.9%) |
|  | |  |
| Seoul National University Bundang Hospital | | 4 (2.6%) |
|  | |  |
| CHA University Bundang Medical Center | | 4 (2.6%) |
|  | |  |
| Sungkyunkwan University Samsung Medical Center | | 12 (7.8%) |
|  | |  |
| Seoul National University Hospital | | 10 (6.5%) |
|  | |  |
| The Catholic University Seoul St. Mary’s Hospital | | 3 (1.9%) |
|  | |  |
| Yonsei University Severance Hospital | | 30 (19.5%) |
|  | |  |
| Ulsan University Asan Medical Center, | | 17 (11.0%) |
|  | |  |
| Ewha Womans University Hospital | | 5 (3.2%) |
|  | |  |
| Dankook University Cheil General Hospital | | 1 (0.6%) |
|  | |  |
| Hanyang University Hospital | | 2 (1.3%) |
|  | | |
